# Optical coherence tomography angiography for the detection of macular neovascularization—comparison of en face versus cross-sectional view

**DOI:** 10.1038/s41433-021-01892-5

**Published:** 2022-01-06

**Authors:** Robert Siggel, Christel Spital, Anna Lentzsch, Sandra Liakopoulos

**Affiliations:** 1 Cologne Image Reading Center, Department of Ophthalmology, University of Cologne, Faculty of Medicine and University Hospital Cologne, Cologne, Germany; 2 Department of Ophthalmology, Helios University Hospital Wuppertal, University of Witten‐Herdecke, Wuppertal, Germany; 3 Department of Ophthalmology, Goethe University, Frankfurt, Germany

**Keywords:** Diseases, Medical research

## Abstract

**Purpose:**

To evaluate sensitivity and specificity of swept source-optical coherence tomography angiography (SS-OCTA) en face images versus cross-sectional OCTA versus a combination of both for the detection of macular neovascularization (MNV).

**Design:**

Prospective cohort study.

**Participants:**

Consecutive patients with various chorioretinal diseases and subretinal hyperreflective material (SHRM) and/or pigment epithelial detachment (PED) on OCT possibly corresponding to MNV in at least one eye.

**Methods:**

102 eyes of 63 patients with fluorescein angiography (FA), OCT and SS-OCTA performed on the same day were included. FA images, the outer retina to choriocapillaris (ORCC) OCTA en face slab, a manually modified en face slab (‘custom slab’), cross-sectional OCTA and a combination of OCTA en face and cross-section were evaluated for presence of MNV.

**Main outcome measures:**

Sensitivity and specificity for MNV detection, as well as the concordance was calculated using FA as the reference.

**Results:**

OCTA en face imaging alone yielded a sensitivity of 46.3% (automated)/78.1% (custom) and specificity of 93.4% (automated)/88.5% (custom) for MNV detection. Cross-sectional OCTA (combination with en face) resulted in a sensitivity of 85.4% (82.9%) and specificity of 82.0% (85.3%). Concordance to FA was *moderate* for automated en face OCTA (*κ* = 0.43), and *substantial* for custom en face OCTA (*κ* = 0.67), cross-sectional OCTA (*κ* = 0.66) and the combination (*κ* = 0.68).

**Conclusion:**

Segmentation errors result in decreased sensitivity for MNV detection on automatically generated OCTA en face images. Cross-sectional OCTA allows detection of MNV without manual modification of segmentation lines and should be used for evaluation of MNV on OCTA.

## Introduction

Macular neovascularization (MNV) is a complication of a variety of chorioretinal diseases such as neovascular age-related macular degeneration, inflammatory disorders, pathological myopia, central serous chorioretinopathy or traumatic rupture of Bruch’s membrane [1–4]. For decades, dye-based fluorescein angiography (FA) has been the gold standard for MNV detection and classification. On FA, MNV has been classified as occult (histologically type 1 MNV) and minimally or predominantly classic (histologically type 2 MNV or mixed type 1 and 2). Additional described subtypes of MNV include retinal angiomatous proliferation (RAP) and polypoidal choroidal vasculopathy (a subtype of type 1 MNV) [5, 6]. Optical coherence tomography (OCT) is preferably used to detect and monitor MNV as well as MNV activity in patients undergoing anti-VEGF therapy [7–11]. OCT-based classification systems of MNV have been proposed, with MNV underneath the RPE being classified as type 1, MNV in the subretinal space presenting as subretinal hyperreflective material (SHRM) as type 2 and RAP as type 3 MNV [6, 10]. However, the distinction between type 1 MNV and a drusenoid PED, as well as between type 2 MNV and other causes of SHRM such as fibrin or acquired vitelliform lesions is not always possible with OCT alone.

Optical coherence tomography angiography (OCTA) allows non-invasive visualisation of retinal and choroidal vessels and is increasingly used to detect MNV lesions [11–19]. OCTA provides three-dimensional (3D) flow information by analysing signal changes between repeated OCTA B-Scans at the same location, presumed to correspond to erythrocyte movements within vessels [14, 15]. Commercially available OCTA systems provide two different options for displaying flow information: the en face view and the cross-sectional view. The en face view is generated by detection of flow information between two segmentation layers [14]. It allows visualisation of blood flow similar to FA as top view mode and enables the separation of depth-related flow information, e.g. the inner and outer retinal vascular plexus, the choriocapillaris and the choroid. Of note, proper segmentation is crucial to avoid image artefacts. Whereas incorrect layer segmentation principally affects quantitative measurements on structural OCT B-scans, it strongly impinges quantitative as well as qualitative evaluation of OCTA en face images [20]. The cross-sectional view provides flow information displayed over a structural OCTA B-Scan allowing detailed correlation of flow signals with structural OCT findings like PEDs or SHRM. Of note, this view is not affected by segmentation errors.

The aim of this study was to compare sensitivity and specificity of automatically provided and manually modified OCTA en face images with cross-sectional OCTA (and their combination) for the detection of MNV secondary to various chorioretinal diseases.

## Materials and methods

For this prospective cohort study, 102 eyes of 63 consecutive patients with SHRM and/or PED on OCT possibly corresponding to MNV secondary to any chorioretinal disease in at least one eye and colour fundus photography (CFP, Canon CX-1 digital retinal camera, Canon, Tokyo, Japan), as well as FA images (HRA2, Heidelberg Engineering, Heidelberg, Germany), performed at the same day were recruited at the Department of Ophthalmology, University of Cologne, Germany.

Patients underwent swept-source (SS)-OCTA imaging with the PLEX^®^ Elite 9000 (Version 1.5.0.15909; Carl Zeiss Meditec, Dublin, CA, USA) for both eyes. The SS-OCTA device has a scanning rate of 100.000 A-scans per second, a central wavelength of 1060 nm and a bandwidth of 100 nm. The full-width at half-maximum axial resolution is ~5 µm in tissue, and the lateral resolution at the retinal surface is ~14 µm. We used a 3 × 3 mm scan raster, which is repeated four times at each position and consists of 300 B-Scans resulting in a distance between section images of 10 µm. The OCTA scan was centred on the fovea. In case the area suspicious for MNV was extrafoveal and not captured, an additional scan was performed displaying the suspected MNV lesion and this scan was used for analysis. The projection artefact removal function was turned off.

Patient data including age, gender, medical history and previous anti-VEGF treatments were collected. A complete ophthalmic examination was performed including slit-lamp and dilated fundus examination, and best-corrected visual acuity was assessed.

The underlying disease and presence of MNV (active or inactive) were evaluated based on multimodal imaging using CFP, FA and OCT masked to OCTA information. Presence of SHRM and/or PED were evaluated on OCT. MNV subtype was graded based on FA (predominantly classic, minimally classic, occult with or without retinal angiomatous proliferation (RAP) and staining scar) by a Reading Center senior grader (RS). OCTA Grading was performed independently by two graders of the Cologne Image Reading Center (CS, RS), masked to all other images of the patient. Discrepancies between graders were solved by open adjudication or discussed with the Reading Center director (SL).

### Grading of en face OCTA

For evaluation of en face OCTA, the automatically by the software generated outer retina to choriocapillaris (ORCC) slab, was used. For this slab, the OCTA software aims to delineate the outer plexiform layer as the inner boundary, and Bruch’s membrane as the outer boundary in order to completely capture a MNV if present.

Since this slab can be affected by segmentation errors one additional manually modified slab (‘custom slab’) was created as follows: the automatically provided ‘RPE-fit’ layer was manually shifted anterior to any possible MNV as inner boundary, as well as posterior to any possible MNV as outer boundary. The aim was to ensure that all areas of possible MNV were captured and included for the generation of the OCTA en face image. The en face slabs were exported from the viewing software and stored in a separate location to ensure masking of graders to all other slabs and imaging modalities during grading.

MNV was defined on en face OCTA images as a flow signal which represents a closed neovascular network with vessel branching *or* a flow signal with a neovascular pattern within the expected foveal avascular zone, not corresponding to the physiological retinal or choroidal vascularisation.

#### Grading of cross-sectional OCTA

For analysis of the cross-sectional OCTA, the grader analysed all OCTA B-scans in the viewing software after selection of the vitreoretinal interface slab to avoid a bias by displaying chorioretinal vessels in the en face view.

Presence of MNV was defined as flow signal within the area of SHRM and/or PED in at least three adjacent OCTA B-scans not considered projection artefacts or pseudoflow. Single pixels which were diffusely spread over the entire PED/SHRM were considered noise and not flow information.

#### Grading of the combination of en face and cross-sectional OCTA

After evaluation of OCTA en face and OCTA cross-sections alone, the combination of both OCTA viewing modes, including the option to manually shift boundaries to create optimised OCTA en face images, was used for MNV evaluation.

#### Evaluation of segmentation errors on OCTA

Presence of segmentation errors in the automatically provided ORCC slab were evaluated for all cases. A clinically relevant segmentation error was considered present, if the automated segmentation failed in at least 12 adjacent OCTA B-scans (corresponding to ~120 µm), resulting in erroneous inclusion of retinal or choroidal vasculature, or partially or complete exclusion of SHRM and/or PED. In these eyes, segmentation errors possibly affect the visualisation of MNV flow information on the corresponding OCTA en face slab, resulting in false positive or false negative evaluation of MNV, or resulting in segmentation artefacts precluding evaluation of MNV.

The study was approved by the local Institutional Review Board and was performed in accordance with the ethical standards of the Declaration of Helsinki. All patients signed an informed consent form.

### Statistics

Data were analysed with IBM SPSS Statistics for Mac, Version 25.0 (IBM Corporation, Armonk, NY, USA). Sensitivity and specificity for MNV detection on OCTA was calculated using FA as the reference. If the quality of images was poor precluding evaluation of MNV, images were graded as ‘cannot grade’. Cases graded as ‘questionable’ or ‘cannot grade’ were grouped with ‘no’ for the statistical analysis. Kappa statistics were used to calculate the concordance between FA and OCTA as well as calculation of intergrader agreement and was classified according to the classification of Landis and Koch [21].

## Results

### Patient demographics

Demographic data and clinical characteristics of the study population are provided in Table 1. Sixty-three patients with SHRM and/or PED on OCT possibly indicating MNV in at least one eye were included in our study. OCTA images for the fellow eye were available in 39 patients, resulting in a total of 102 eyes available for analysis. A total of 80 eyes (78.4%) demonstrated SHRM and/or PED on OCT, and MNV was detected on FA in 41 cases (40.2%).

**Table 1:** Demographic data and patients characteristics.

Parameter		*n*	%
Number of eyes		102	100
Number of patients		63	100
Gender (male/female)		33/30	52.4/47.6
Age, mean ± SD, years (range)		62.2 ± 18.9	(19–91)
Presence of MNV suspicious lesions on OCT			
	PED and/or SHRM	80	78.4
	PED	76	74.5
	SHRM	44	43.1
Presence of MNV on FA			
	Any MNV	41	40.2
	Predominantly classic MNV	15	36.6
	Minimally classic MNV	3	7.3
	Occult MNV without RAP	9	21.9
	Occult MNV with RAP	2	4.9
	Staining Scar	12	29.3
Diagnosis (multimodal imaging)			
	AMD	43	42.2
	Chorioretinitis	11	10.8
	CSCR	10	9.8
	High myopia	7	6.9
	Pattern dystrophy	5	4.9
	Acquired vitelliform lesions	3	2.9
	Other chorioretinal disease	17	16.7
	No pathology	6	5.9

*AMD* *age-related macular degeneration, MNV* *macular neovascularization, CSCR* *Central Serous Chorioretinopathy, FA* *fluorescein angiography, PED* *pigment epithelial detachment, RAP* *retinal angiomatous proliferation, SD* *standard deviation, SHRM* *subretinal hyperreflective material, OCT* *optical coherence tomography.*

### Sensitivity and specificity of OCTA in reference to FA

Using the automatically provided ORCC slab, sensitivity for detection of MNV was 46.3% and specificity was 93.4% (Table 2). The custom slab showed a sensitivity of 78.1% and specificity of 88.5%. The evaluation of cross-sectional OCTA resulted in a sensitivity of 85.4% and specificity of 82%. By combining en face and cross-sectional OCTA, MNV was identified with a sensitivity of 82.9% and specificity of 85.3%.

**Table 2:** Sensitivity and specificity of en face OCTA, cross-sectional OCTA and the combination of both for MNV detection in reference to FA.

OCTA viewing mode	Sensitivity	Specificity	MNV Questionable	MNV Cannot Grade	Concordance^a^ to FA	Intergrader Agreement
En face OCTA (ORCC, automatic segmentation)	46.3%	93.4%	19.6%	4.9%	*κ* = 0.43	*κ* = 0.69
En face OCTA (manually modified segmentation)	78.1%	88.5%	10.8%	0%	*κ* = 0.67	*κ* = 0.86
Cross-sectional OCTA	85.4%	82.0%	2%	0%	*κ* = 0.66	*κ* = 0.91
Combination	82.9%	85.3%	5.9%	0%	*κ* = 0.68	*κ* = 0.87

*OCTA* *optical coherence tomography angiography, ORCC* *outer retina to choriocapillaris. FA* *fluorescein angiography, MNV* *macular neovascularization*

^a^Criteria according to Landis and Koch: poor (kappa < 0.00), slight (0.00–0.20), fair (0.21–0.40), moderate (0.41–0.60), substantial (0.61–0.80), almost perfect (0.81–1.00)

Concordance to FA was *moderate* for automatically segmented en face OCTA (*κ* = 0.43), and *substantial* after manual modification of the segmentation (*κ* = 0.67), for cross-sectional OCTA (*κ* = 0.66) and the combination (*κ* = 0.68). Intergrader agreement was *almost perfect* for cross-sectional OCTA alone (*κ* = 0.91), the combination of en face and cross-sectional OCTA (*κ* = 0.87) and the en face custom slab alone (*κ* = 0.86). Automatically segmented en face OCTA yielded a *substantial* intergrader agreement (*κ* = 0.69) (Table 2).

#### Segmentation errors

**Fig. 1:**
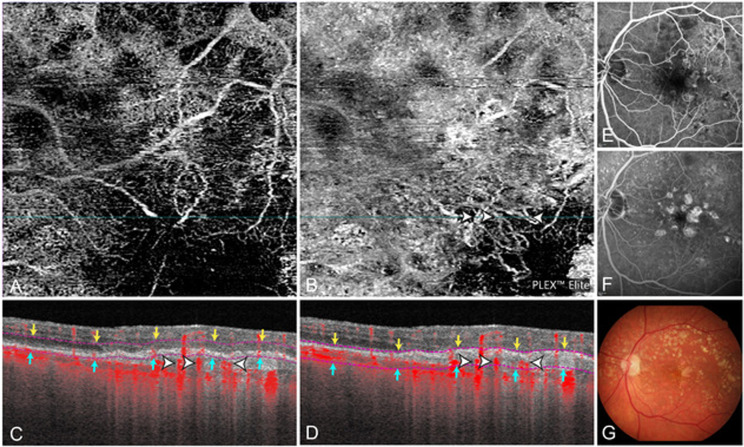
Example case demonstrating severe segmentation errors in the automatically provided ‘ORCC’ en face image resulting in false-negative MNV evaluation. En face image of the automatically provided ‘ORCC’ slab (**A**). The corresponding cross-sectional OCTA shows sub-RPE flow information (*red* pixel with *white* arrows) posterior to the ORCC segmentation borders (*magenta* dashed lines) (**C**). By manually shifting the inner and outer segmentation borders (**D**), the MNV becomes visualised (*white* arrow) on OCTA en face (**B**). Corresponding fluorescein angiography shows window defects but no active MNV leakage (**E** mid phase **F** late phase). Corresponding colour fundus photography shows drusen and hypopigmentation (**G**).

**Fig. 2:**
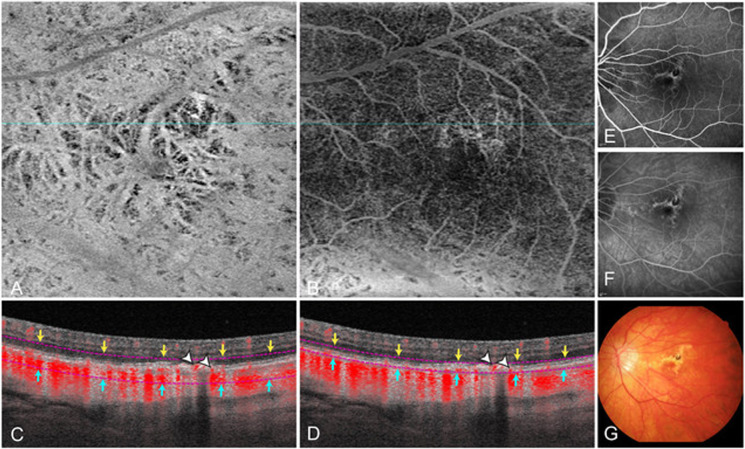
Example case demonstrating segmentation errors in the automatically provided ‘ORCC’ en face image resulting in false-negative MNV evaluation. En face image of the automatically provided ‘ORCC’ slab (**A**). The corresponding cross-sectional OCTA shows sub-RPE flow information (*red* pixel) of the MNV (*white* arrows) (**C**). When correcting the segmentation lines (**D**), the MNV becomes more easily visible on OCTA en face imaging (**B**). Corresponding fluorescein angiography shows a staining scar (**E** mid phase, **F** late phase) and the colour fundus photography (**G**) a yellowish fibrotic lesion.

Clinically relevant segmentation errors were considered to be present in the ORCC slab in 38% (39/102) of cases (Figs. 1, 2). Out of those, SHRM was at least partially excluded by the segmentation lines in 44% (17/39) and a PED was at least partially excluded in 56% (22/39), thus in those cases MNV was potentially not or incompletely visualised on the ORCC en face images.

## Discussion

MNV is a severe complication of various chorioretinal diseases, however, identification of MNV may be challenging. While FA was the gold standard for MNV detection for decades, non-invasive OCTA gained importance for visualisation of MNV lesions by rising experience with this relative new technique [14]. OCTA allows to visualise, characterise, monitor and quantify the vascular network of MNV membranes in co-registration with the structural OCT [13, 14, 16, 22]. MNV detection on OCTA has been compared to FA by various authors, reporting sensitivity values between 50% and 100% for detection of MNV [17, 23–27]. These studies differed regarding the underlying disease, MNV subtypes, OCTA instruments, evaluated automatically provided slabs, approach of manual modification of slabs (if performed) as well as masking to other imaging modalities. Some studies used only the OCTA en face view without corresponding cross-sectional OCTA [26]. Other studies did not report whether the cross-sectional view was taken into account in addition to OCTA en face images [17, 25].

Our results indicate a clear improvement of sensitivity in detection of MNV by evaluation of cross-sectional OCTA compared to the automatically provided ORCC en face slab alone, and a slight improvement compared to manually modified OCTA en face slabs. By combining both en face and cross-sectional OCTA, the sensitivity did not increase further in our study. Therefore, our findings implicate the benefit of taking cross-sectional OCTA B-scans with flow overlay into account for evaluation of MNV in neovascular AMD or other diseases. This is in line with the findings of Faridi et al., who found a sensitivity of 97.5% and specificity of 100% by combining en face and cross-sectional imaging in neovascular AMD patients [11]. In contrast to our work, Faridi et al achieved a much higher detection rate of MNV using OCTA en face images alone with a sensitivity of 81.3% compared to our results with a sensitivity of 46.3%. Differences between our study and the study performed by Faridi et al include the use of different OCTA imaging systems, differences in patient population regarding the underlying diseases and methodology to define the gold standard for statistical analysis of sensitivity and specificity, In addition, Faridi et al. used all provided en face slabs (whole scan, superficial retina, outer retina and choriocapillaris) to evaluate the presence of MNV, whereas we only used the ORCC slab and a customised slab. However, Faridi et al. did not analyse the sensitivity and specificity of cross-sectional OCTA masked to en face slabs.

There are some advantages of the en face view. Most of the clinicians are familiar with the en face view as ‘top view’ mode because of the similarity to FA images. Typical MNV morphological patterns may be detectable with a glance in many cases. The growth patterns can be easily monitored over time and quantitative measurements can be conducted [18, 22, 28]. Nevertheless, as we could show, the sensitivity of MNV detection is low and depends widely on correct layer segmentations and the incidence of image artefacts. Our study group as well as others could previously show that sensitivity for MNV detection on en face OCTA increases with optimised manual segmentation to completely include any SHRM and/or PED considered suspicious for MNV [29]. However, even manually modified OCTA en face images yielded lower sensitivity values compared to cross-sectional OCTA [29]. One reason for difficulties in evaluating MNV based on en face OCTA alone may be the lack of information regarding the location of the suspected MNV within the en face image. Multimodal imaging such as OCT or FA in addition to en face OCTA images may provide this information and thus improve sensitivity.

**Fig. 3:**
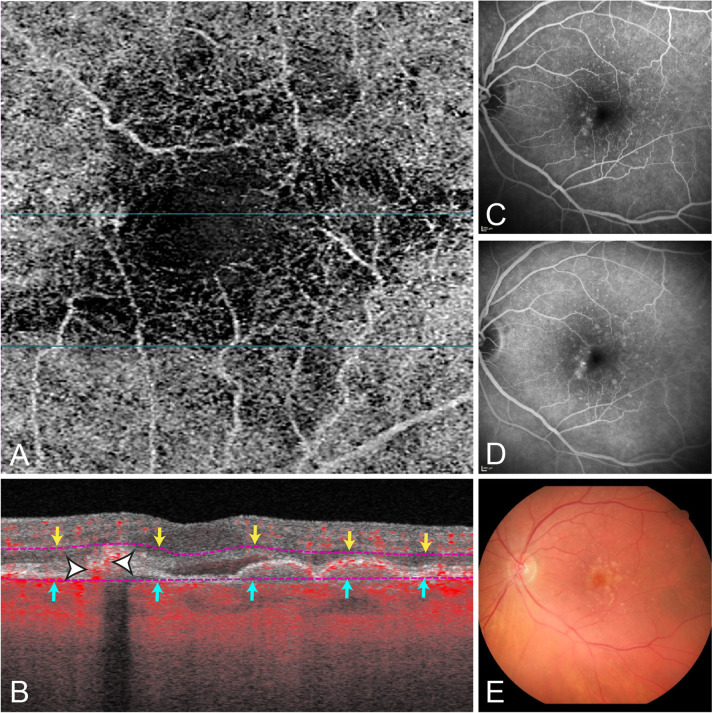
Example case of MNV graded as ‘questionable’ present on the en face OCTA. En face image of the automatically provided ‘ORCC’ slab(**A**). Cross-sectional OCTA shows definite flow in the sub-RPE space (*red* pixel) and was graded as MNV present (*white* arrows) (**B**). Corresponding fluorescein angiography shows an active occult MNV leakage (**C** mid phase, **D** late phase). Colour fundus photography (**E**) reveals a small yellowish-brown lesion and multiple confluent drusen.

The advantage of the cross-sectional OCTA view is the direct correlation of the flow signal to the structural OCT, also this viewing mode gives additional depth-information (Fig. 3). Overlay of flow information on cross-sectional OCTA B-scans is independent of layer segmentations and therefore very useful in cases of severe segmentation errors. In contrast to en face OCTA, none of the cross-sectional OCTA cases were evaluated as ‘cannot grade’, which additionally indicates the higher confidence in MNV evaluation by graders. However, for evaluation of the cross-sectional view, the examiner has to scroll through a large number of OCTA B-scans, which may be time consuming in daily routine. Another disadvantage is that the morphological pattern of MNV is insufficiently evaluable in this viewing mode. (24) Automated projection artefact removal (PAR) provided by the software do not affect the cross-sectional view, therefore the evaluation of flow information in MNV suspicious lesions and the differentiation to projection artefacts of e.g. retinal vessels may sometimes be difficult. Moreover, there may be imaging artefacts such as pseudoflow within hyperreflective material such as hard exudates, drusen or SHRM [30]. PAR reduces but does not eliminate pseudoflow, and pseudoflow may be detected within the foveal avascular zone, thus some authors concluded that other factors, such as Z-axis micromotion, may also contribute to pseudoflow [30]. Care should be taken to not misinterpret these artefacts as indicators for the presence of MNV.

Limitations of our study include the small sample size. However, our study has several strengths including the use of consecutive patients with a variety of chorioretinal diseases, the masked evaluation of various imaging modalities as well as the evaluation of images by Reading Center graders.

In conclusion, our study demonstrates that analysing cross-sectional OCTA yields high sensitivity and specificity in the detection of MNV in a variety of chorioretinal diseases and improves diagnostic accuracy compared to en face OCTA images alone. Manually modified segmentation lines are necessary to improve detection sensitivity of MNV compared to the automatically provided ORCC slab. Cross-sectional OCTA is independent of the position of segmentation lines and may improve accuracy of image evaluation compared to en face images alone by identifying the MNV-suspicious area and without time-consuming manual correction of segmentation errors in daily routine.

### Summary

#### What was known before

- Both cross-sectional OCTA and en face OCTA can be used for MNV analysis. It is still unclear which has the higher diagnostic accuracy for detecting macular neovascular membranes (MNV).

#### What this study adds

- Cross-sectional OCTA yields high sensitivity and specificity in the detection of MNV in a variety of chorioretinal diseases and improves diagnostic accuracy compared to en face OCTA images alone.
